# Animal Experimental Study on Delayed Implantation in a Severely Atrophic Alveolar Ridge Reconstructed Using a 3D-Printed Bioactive Glass Scaffold: A Pilot Study

**DOI:** 10.3390/jfb16050176

**Published:** 2025-05-13

**Authors:** Lei Deng, Liya Ai, Runxu Li, Wusheng Xu, Lingling Zheng, Chao Wang, Haitao Huang

**Affiliations:** 1 Department of Stomatology, The First Affiliated Hospital of Dalian Medical University, Dalian 116011, China; denglei12987@163.com (L.D.); lirunxu1997@outlook.com (R.L.); x17866525175@163.com (W.X.); 2 Key Laboratory of Biomechanics and Mechanobiology, Ministry of Education, Beijing Advanced Innovation Center for Biomedical Engineering, School of Biological Science and Medical Engineering, School of Engineering Medicine, Beihang University, No. 37, Xueyuan Road, Beijing 100083, China; elia1212@163.com (L.A.); zhenglingling325@163.com (L.Z.); 3 State Key Laboratory of Virtual Reality Technology and Systems, Beihang University, No. 37, Xueyuan Road, Beijing 100083, China

**Keywords:** guided alveolar bone regeneration, 3D printing, A-W bioactive glass, dental implant

## Abstract

In this study, a scaffold was designed using 3-Matic software 12.0 (Materialise, Leuven, Belgium) and fabricated via Digital Light Processing (DLP) 3D printing technology, followed by a mechanical property evaluation. The scaffold was bilaterally implanted into mandibular bone defect models in four Beagle dogs to facilitate guided alveolar bone regeneration. After 12 weeks, samples were harvested from two dogs for radiographic and histopathological evaluations. In the remaining two dogs, two dental implants were placed into the scaffold sites. After an additional 12 weeks, samples were harvested for further radiographic and histopathological assessments. (1) Compression testing of the scaffold demonstrated a compressive strength of 24.77 ± 2.36 MPa. (2) Three of the implantation sites exhibited poor wound healing and exposure of the bone grafts early post-surgery (4 weeks), with an exposure rate of 37.5%. (3) Micro-CT imaging revealed a uniform distribution of newly formed bone within the scaffold, with an average bone height of 4.05 ± 0.55 mm and a bone volume fraction of 43.93 ± 4.68%. Histopathological analysis demonstrated the presence of vascularized tissue, non-calcified bone, and newly calcified bone within the scaffold. Additionally, newly formed calcified bone and vascularized tissue were observed at the interface between the implant and the scaffold. These findings suggest that DLP 3D-printed A-W bioactive glass scaffolds represent a promising approach for guided alveolar bone regeneration in dental implant applications.

## 1. Introduction

Tooth loss is a common clinical condition, and dental implants have become the primary modality for tooth restoration due to their distinct advantages [1,2]. The success of implant therapy is highly dependent on the availability of sufficient and stable bone at the implantation site. Therefore, promoting new bone regeneration in severely atrophic alveolar ridges has become a central focus of current research [3]. In traditional guided bone regeneration (GBR) techniques, autologous bone grafts or bone graft substitutes are primarily used to augment horizontal or vertical bone volume in alveolar ridge defects. Autologous bone grafts are regarded as the “gold standard” for GBR because of their osteogenic, osteoinductive, and osteoconductive properties, and their lack of immunogenicity [4,5]. However, the harvesting of autologous bone requires an additional surgical site, increasing operative time, patient trauma, and the risk of infection [6,7]. Moreover, autologous bone may not conform precisely to the defect morphology, potentially leading to early resorption [8].

Bone graft substitutes such as allogeneic and xenogeneic bone particles circumvent the drawbacks associated with harvesting autologous bone. Nevertheless, these substitutes pose risks of immune rejection and disease transmission. Additionally, loose particulate grafts often require barrier membranes, such as bio-collagen membranes or titanium meshes, to maintain the osteogenic space [9,10]. Bio-collagen membranes, which are absorbable and widely used due to their biocompatibility, degradability, and affordability, provide limited space-maintaining ability in extensive alveolar bone defects [11,12]. In contrast, non-absorbable barrier membranes, such as polyether ether ketone (PEEK) meshes and titanium meshes, offer superior space maintenance [10,13,14,15]. However, their clinical application is frequently limited by challenges in secondary removal, suboptimal osteogenic outcomes, and complications such as mesh exposure and infection [16,17]. To overcome these limitations, ongoing research is focused on developing advanced synthetic bone graft substitutes.

With progress in the structural and functional understanding of bioceramics and their mechanisms in tissue repair, numerous bioceramic products have been developed and have shown considerable promise in tissue regeneration. Bioceramics can be classified into four categories: bioinert ceramics, bioactive ceramics, resorbable bioceramics, and bioceramic composites. Among these, bioactive glass constitutes a notable subgroup of bioactive ceramics. Owing to its excellent bioactivity and the ability to form a stable chemical bond with bone, bioactive glass has garnered significant attention from the international biomaterials community since its inception [18,19]. Bioactive glass is typically categorized into four types: melt-derived bioactive glass, bioactive microcrystalline glass, sol-gel-derived bioactive glass, and nanoscale bioactive glass. As an emerging scaffold material in bone tissue engineering, bioactive glass exhibits substantial application potential.

However, alveolar bone possesses complex anatomical morphology, with each surface characterized by unique free-form features. While bioactive glass can serve as a scaffold material for GBR, it may not precisely conform to the geometry of the bone defect, thereby compromising regenerative outcomes [20]. The advent of 3D printing technology provides a highly accurate fabrication method for bioceramic materials, enabling optimal morphological adaptation between the scaffold and the defect site.

The rapid advancement of 3D printing has led to the development of several widely utilized techniques, including three-dimensional printing (3DP), selective laser melting/sintering (SLM/SLS), direct ink writing (DIW), inkjet printing (IJP), digital light processing (DLP), fused deposition modeling (FDM), stereolithography (SL), and laminated object manufacturing (LOM). These technologies have been extensively explored for the fabrication of bioactive materials [21,22,23,24,25]. Among them, DLP has emerged as a prominent technique for 3D printing bioactive scaffolds, as it enables the high-resolution fabrication of biomaterials. Its utility in oral and craniofacial tissue regeneration has been previously demonstrated [26,27].

The use of 3D-printed patient-specific bone graft materials not only reduces material waste, but also enhances surface properties and porous structures, thereby promoting cell adhesion, migration, proliferation, and osteogenic differentiation—features essential for successful GBR. Furthermore, this approach significantly reduces surgical operation time in clinical settings [28]. Various studies have explored the use of 3D printing for fabricating personalized bone grafting materials [29]. In the biomedical field, 3D-printed bioactive ceramics composed of hydroxyapatite/tricalcium phosphate (HA/TCP) have shown encouraging preliminary outcomes in guiding alveolar bone regeneration [30,31]. Nevertheless, the design and fabrication of bioactive ceramic scaffolds that precisely match alveolar bone defects while enabling successful implant placement and functional occlusion using computer-aided design/manufacturing (CAD/CAM) or 3D printing technologies remains a major challenge. Moreover, it is still uncertain whether these scaffolds can support osseointegration and withstand functional loading.

A review of the literature indicates that research on 3D-printed bioactive glass scaffolds in this context is limited, particularly concerning alveolar bone regeneration. A-W bioactive microcrystalline glass used in the present study theoretically demonstrates superior performance and osteogenic potential compared to previously reported HA/TCP-based scaffolds. This study aims to evaluate the performance and osteogenic capacity of an A-W bioactive microcrystalline glass scaffold (A-W bioactive glass, A-W BG) fabricated using DLP 3D printing technology in promoting alveolar bone regeneration in Beagle dogs. Furthermore, this study assesses whether dental implants placed into the scaffold after 12 weeks of healing could achieve successful osseointegration. This research successfully achieved implant placement following guided bone regeneration using 3D-printed bioactive glass scaffolds, representing the first report of its kind in this field.

## 2. Materials and Methods

This study included four healthy adult male Beagle dogs, each approximately 12 months old, with a body weight of 12–14 kg (Laboratory Animal Qualification Certificate Number: SYXK [Liao] 2018-0007). The study protocol was approved by the Animal Research Ethics Committee of Dalian Medical University (Ethics Approval Number: AEE23036). Prior to the commencement of the experiment, all Beagle dogs underwent a one-week acclimatization period to the experimental environment and were provided with a controlled daily diet and water intake.

### 2.1. Design and Fabrication of 3D-Printed Bioactive Glass Scaffolds

**Figure 1:**
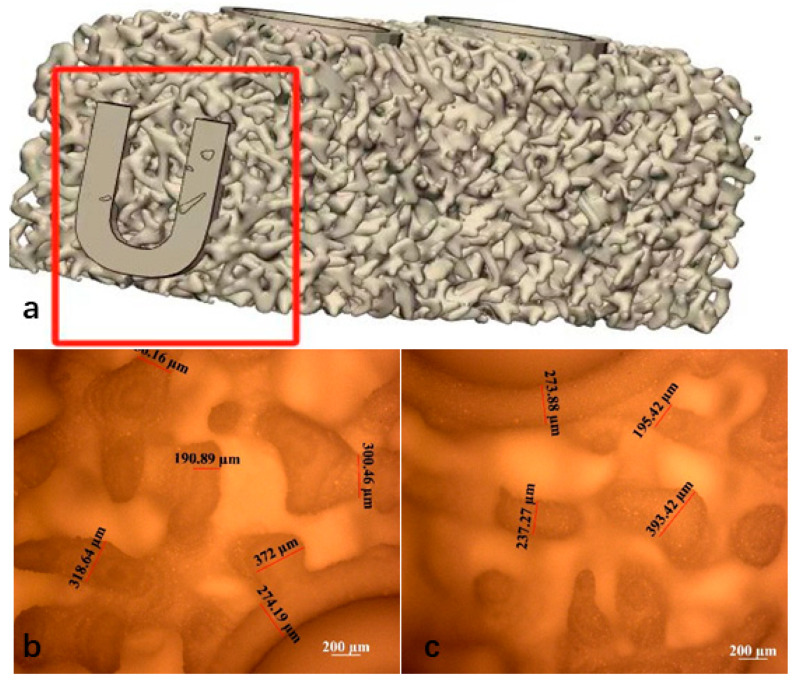
(**a**) Uniform trabecular bone density design of the scaffold (Mark the “U” mark). Sintered scaffold material after de-binding (**b**) top view, (**c**) bottom view.

The 3D-printed scaffolds in this study were designed using 3-Matic software (Materialise, Leuven, Belgium) and exported as standard tessellation language (STL) files. The scaffold model was designed with dimensions of 15 mm × 5 mm × 5 mm and was filled with a trabecular bone-mimicking structure. Two fixation holes were incorporated into the design, and the trabecular bone density was uniformly distributed, as illustrated in Figure 1. The average porosity of the scaffold was 86.37%, while the scanned porosity after sintering was 68.78%. The average pore size of the scaffold was 220 μm before sintering and 180 μm after sintering.

The resin material used in this experiment was supplied by Shanghai Aladdin Biochemical Technology Co., Ltd., Shanghai, China. The A-W bioactive glass powder, with a particle size of 20 μm, was provided by the Naton Institute of Medical Technology, Beijing, China. The A-W bioactive glass powder was mixed with a photosensitive resin and subsequently ball-milled for 1–2 h to obtain a photosensitive resin-ceramic slurry with a solid content of approximately 52%, which was used as the printing material.

The slurry was then processed using a 3D printer employing DLP technology (Technology and Engineering Center for Space Utilization, Chinese Academy of Sciences, Beijing, China). The printing process was conducted based on STL files, with an exposure time of 3 s, a layer thickness of 30 μm, and a light wavelength of 405 nm. Upon completion of the curing, the scaffold was carefully removed from the curing platform and subjected to ultrasonic cleaning in distilled water for 20 min to eliminate any uncured slurry remaining on the model surface. The scaffolds were sintered according to the following temperature profile: initially, the temperature was increased at a rate of 2 °C/min until reaching 600 °C, where it was maintained for 120 min. Subsequently, the temperature was further increased at a rate of 1 °C/min until reaching 1100 °C, where it was held for another 120 min before being allowed to cool naturally to room temperature. This process resulted in the formation of sintered bioactive glass scaffolds, with a post-sintering shrinkage rate of approximately 22%. Microscopic observation and measurement of the sintered scaffolds revealed a printing accuracy of approximately 300 μm, with a maximum precision of up to 190 μm. The specific morphology of the scaffolds is presented in Figure 1b.

### 2.2. Mechanical Performance Testing of 3D-Printed Bioactive Glass Scaffolds

The mechanical compressive properties of the scaffolds were evaluated at a controlled room temperature of 25 °C using a universal mechanical testing machine (SHIMADZU AGS-X, Kyoto, Japan). A total of six samples were tested. The porous specimens (5 mm × 5 mm × 5 mm) were secured onto the base fixture, and a unidirectional vertical compressive deformation was applied by the upper platen at a rate of 0.5 mm/min until structural failure occurred. The compressive strength (σ) was then calculated using the following formula, $\sigma =\frac{F}{A}$, where σ represents the compressive strength (MPa), F is the maximum compressive load sustained by the specimen (N), and A denotes the cross-sectional area of the specimen perpendicular to the loading direction (mm^2^). A stress–strain curve was also plotted.

**Figure 2:**
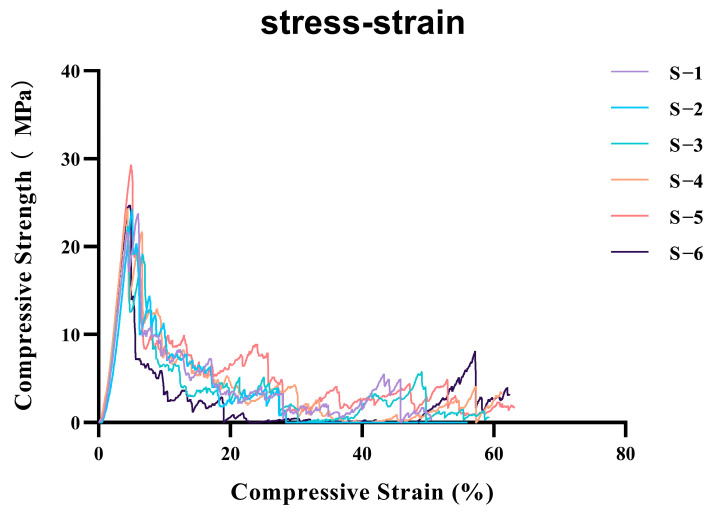
Compressive stress-strain curves of six parallel specimens (labeled S−1 to S−6) under uniaxial compression.Mean compressive strength: 24.77158; standard deviation: 2.359387; coefficient of variation: 9.5246%. (Colored lines represent individual replicates tested at a strain rate of 0.5 mm/min).

The compression tests for the A-W bioactive glass (A-W BG) scaffolds with a homogeneous density design yielded a compressive strength of 24.77 ± 2.36 MPa, with a coefficient of variation of 9.5246% (Figure 2).

### 2.3. Surgical Procedures

**Figure 3:**
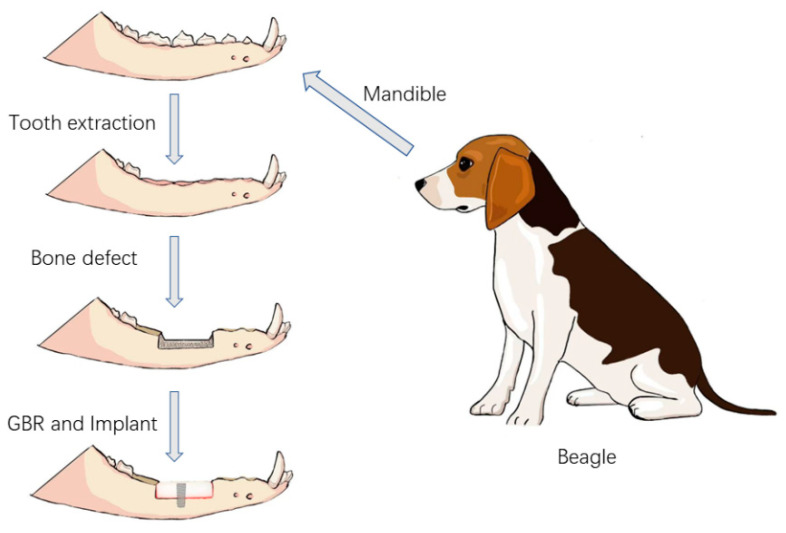
Schematic diagram of the surgical procedure.

The experimental surgical design was divided into four stages (Figure 3).

Stage 1: Four Beagle dogs, numbered 01–04, underwent general anesthesia, followed by the extraction of their bilateral mandibular premolars (P1–P4) and the first molar (M1).

Stage 2: After an 8-week healing period of the alveolar bone, a bone defect model was created in the edentulous mandibular segment of each Beagle dog using an osteotomy guide plate. The 3D-printed bioactive glass scaffolds were then fixed in the bone defect areas on both sides of the mandible.

Stage 3: After 12 weeks of bone healing, two Beagle dogs (numbered 01 and 03) were selected for radiographic and histopathological evaluations of the surgical area. The remaining two Beagle dogs (numbered 02 and 04) underwent the implantation of two dental implants into the bioactive glass scaffolds.

Stage 4: Twelve weeks after implantation, the bioactive glass scaffold material, along with the surrounding mandibular bone containing the implants, was harvested for radiographic and histopathological evaluations.

#### 2.3.1. Medication and Anesthesia

All experimental dogs were subjected to fasting, including food and water restriction, for 12 h before surgery. Prior to anesthesia, cephalexin tablets (Vicq Co., Ltd., Nice France) were administered orally (PO). Intramuscular (IM) injections of butorphanol tartrate (Jiangsu China National Pharmaceutical Group Beikang Pharmaceutical Co., Ltd., Taizhou, China) were administered, followed by intravenous (IV) injections of propofol (Guangdong Jiabo Pharmaceutical Co., Ltd., Qingyuan, China). Anesthesia was then induced through the inhalation of a mixture of oxygen (O_2_) and isoflurane (Hebei Jinda Fu Pharmaceutical Co., Ltd., Xingtai, China).

Throughout the surgical procedure, heart rate, oxygen saturation, and body temperature were continuously monitored. Additionally, prior to any surgical manipulation of the mandibular alveolar ridge on each side, local infiltration anesthesia was performed using lidocaine. Postoperatively, all of the experimental dogs received intramuscular injections of Baytril (Bayer AG, Leverkusen, Germany) at a dose of 0.1 mL/kg, along with Puyikang injectable cefquinome sulfate (Henan Zhongsheng Animal Pharmaceutical Co., Ltd., Zhengzhou, China) at a dose of 10 mg/kg, for a total of five days, to prevent inflammation.

#### 2.3.2. Tooth Extraction

**Figure 4:**
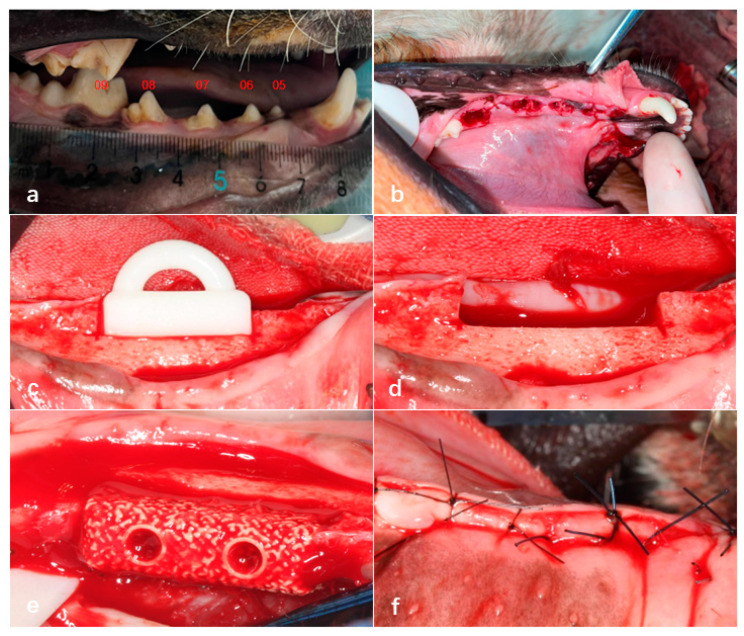
Extraction of teeth in Beagle dogs 05–09 under general anesthesia (**a**,**b**). Creation of bone defects in the edentulous mandibular segments of Beagle dogs using an osteotomy guide plate (**c**,**d**), fixation of bioactive glass scaffolds in the bone defect areas using titanium screws (**e**), and tight suturing of the gingival incision (**f**).

Initially, a full-mouth cleaning was performed on all of the experimental dogs using an ultrasonic scaler. After disinfection with povidone–iodine, a gingival separator was used in the surgical area from the premolars to the first molar to detach the gingiva and expose the alveolar ridge. A fissure bur was then employed to section each tooth requiring extraction (excluding the first premolar) into two separate roots. A dental elevator was inserted between the tooth roots and the alveolar bone to loosen each root, after which dental forceps were used to extract them individually. Following extraction, the alveolar sockets were curetted to remove residual fragments and irrigated with sterile saline. Finally, the gingiva was sutured using 3-0 absorbable sutures. Each experimental dog underwent the extraction of five teeth on one side of the mandible (four mandibular premolars and the first mandibular molar) (Figure 4a,b).

#### 2.3.3. Preparation of Bone Defect Sites and Bioactive Glass Implantation

Eight weeks after tooth extraction, a bone defect model measuring 15 mm in length, 5 mm in width, and 5 mm in height was created in the edentulous mandibular segments of Beagle dogs using an osteotomy guide plate. Subsequently, 3D-printed bioactive glass scaffolds with a homogeneous density design were fixed in the bone defect areas on both sides of the mandible using fixation screws. The gingiva was then sutured tightly with 3-0 absorbable sutures (Figure 4c–f).

After 12 weeks of bone healing, a gingival flap was elevated in the grafted areas on both sides of the mandible to expose the implanted bioactive glass scaffolds, allowing for the observation of newly formed bone. Two Beagle dogs (numbered 01 and 03) were euthanized, and their mandibles, along with the bioactive ceramic scaffolds, were harvested as experimental specimens. All specimens were fixed in 10% neutral-buffered formalin for subsequent radiographic and histopathological evaluations.

For the remaining two Beagle dogs (numbered 02 and 04), two dental titanium alloy implants measuring 7 mm in length and 2 mm in diameter (Leiden Company, Beijing, China) were inserted at the original fixation screw positions in the bioactive glass scaffolds. The gingiva was then sutured tightly without tension. Twelve weeks after implant placement in the bioactive glass scaffolds, the mandibles containing the implants, along with the surrounding bone, were harvested and fixed in 10% neutral-buffered formalin for subsequent radiographic and histopathological evaluations.

#### 2.3.4. Radiographic Analysis

Prior to specimen collection, all experimental sites were subjected to X-ray micro-computed tomography (micro-CT) scanning using the Skyscan 1173 system (Bruker-micro CT, Belgium) to evaluate the healing status between the 3D-printed bioactive glass scaffolds and the bone bed. Bone formation capacity was assessed using Micro-CT scanning (Viva CT40; Scanco Medical AG, Bassersdorf, Switzerland).

The scanning parameters were set to an X-ray voltage of 70 kV with a resolution of 19 μm. The scanned data were exported in DICOM format for storage. The DICOM data were reconstructed and further analyzed using Mimics Research software (version 21.0). The newly formed bone within the region of interest and the scaffold were initially segmented by adjusting different CT grayscale thresholds. Further differentiation was achieved by capturing and registering micro-CT images that corresponded with similar histological images, allowing for the quantification of the percentage of new bone within the bioactive glass scaffolds.

### 2.4. Histopathological Evaluation

All specimens were fixed in 10% neutral-buffered formalin at 4 °C for one week, followed by dehydration and paraffin embedding. Histological slides were prepared by making longitudinal paraffin sections of the bone tissue. Eight slides were stained using the methylene blue-picro fuchsin staining method, and the newly formed calcified bone within the scaffold materials was examined under a microscope.

## 3. Statistical Analysis

Statistical analyses were conducted using the Statistical Package for the Social Sciences (SPSS, version 12.0K; SPSS Inc., Chicago, IL, USA). Quantitative data are presented as mean ± standard deviation and median. The Shapiro–Wilk test was used to assess normal distribution. If significance (*p* < 0.05) is presented, it means that the data do not satisfy normal distribution and vice versa.

## 4. Results

### 4.1. Microstructure Evaluation

**Figure 5:**
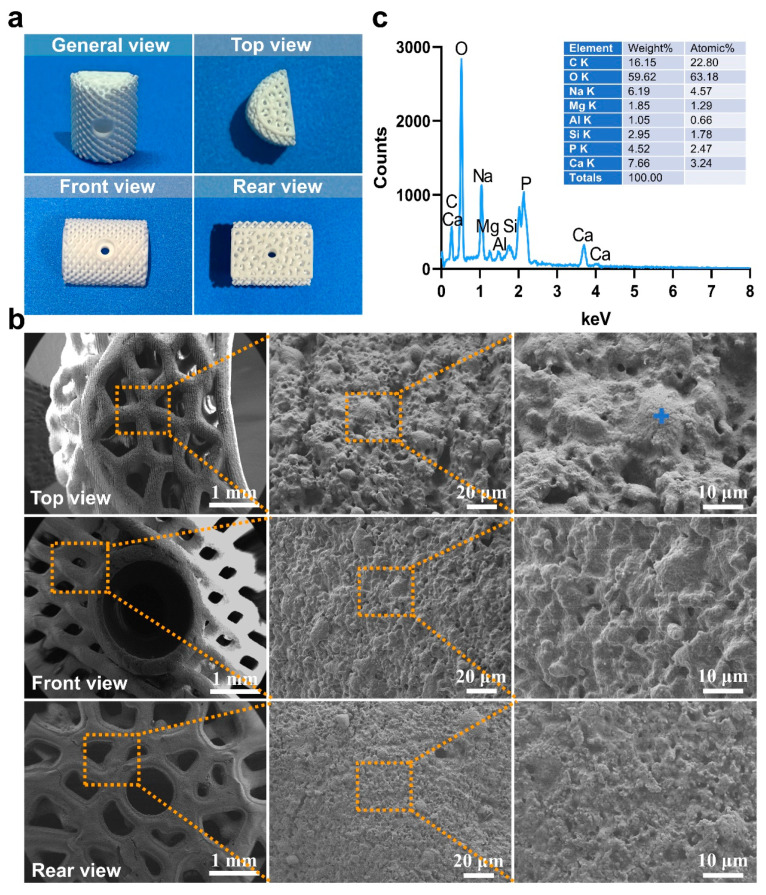
(**a**) The macro morphology of bioactive glass materials used in this experiment under different viewing angles, (**b**) The microstructure morphology of the material was observed in selecting the dotted line positions under various perspectives (**c**) The energy-dispersive spectroscopy (EDS) results (indicated by blue markers).

The macroscopic and microscopic morphology of the 3D-printed bioactive glass scaffold is systematically illustrated in Figure 1. Macroscopically, the scaffold exhibits a hierarchically porous architecture with interconnected pores (100–500 μm diameter), a critical feature for nutrient diffusion and osteogenic cell infiltration (Figure 5a). At higher magnification, the microstructure reveals a homogeneous glass matrix with localized nanocrystalline phases (~50–200 nm), indicative of controlled sintering during fabrication (Figure 5b). Energy-dispersive X-ray spectroscopy (EDS) mapping (blue highlights) confirms the predominant presence of Si, Ca, P, and Na—key elements for bioactivity—with a Ca/P ratio of ~1.6, closely resembling natural hydroxyapatite (Figure 5c). This compositional profile aligns with recent advancements in China (e.g., ShanghaiTech’s SiO_2_-CaO-P_2_O_5_ systems) and global research (e.g., EU-funded GLACIER project) that optimize ion release kinetics for bone regeneration.

### 4.2. Clinical Evaluation

**Figure 6:**
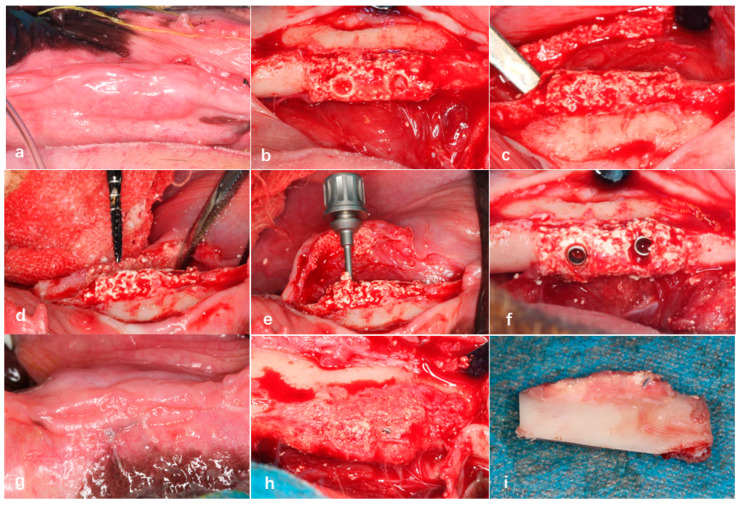
The mucosal healing of the gingiva was favorable in the non-exposed group (**a**). After gingival flap reflection, the bioactive glass scaffold was well integrated with the alveolar bone defect area, with new bone formation visible inside the scaffold (**b**) and at the junction with the defect area (**c**). Preparation of implant osteotomies in the bioactive glass scaffolds (**d**), placement of two dental implants (**e**,**f**). Twelve weeks after implant placement, gingival mucosal healing was favorable (**g**), and the implant showed “bone healing” with new bone coverage on the surface (**h**,**i**).

During the 32-week experimental period, a total of four Beagle dogs were included in the study, with eight surgical sites designed for implantation of bioactive glass scaffolds (homogeneous trabecular density design) in the bilateral mandibular bone defect areas of the dogs. No deaths were reported among the experimental dogs during the follow-up. Three of the implantation sites exhibited poor wound healing and exposure of the bone grafts early post-surgery (4 weeks), with an exposure rate of 37.5%. At 12 weeks post-surgery, after gingival flap reflection, significant soft tissue presence was observed between the scaffold material and the bone defect area, and the scaffold material exhibited mobility. In the remaining five non-exposed scaffold sites, bone healing was observed radiographically at the interface between the scaffold base and the alveolar bone. After gingival flap reflection, no mobility was observed in the scaffold material, and new bone formation was visible at the junction between the scaffold material and the defect area (Figure 6a–c).

In the Beagle dogs where the dental implants were placed on the bioactive glass scaffolds 12 weeks post-surgery, gingival mucosal healing was favorable, with no exposure of the bioactive scaffold material. After gingival flap reflection, the bioactive glass scaffolds implanted with dental implants showed good bone healing with the alveolar bone defect area, and the implants exhibited new bone coverage, demonstrating stable implant integration without loosening (Figure 6d–f).

### 4.3. Radiographic Evaluation

**Figure 7:**
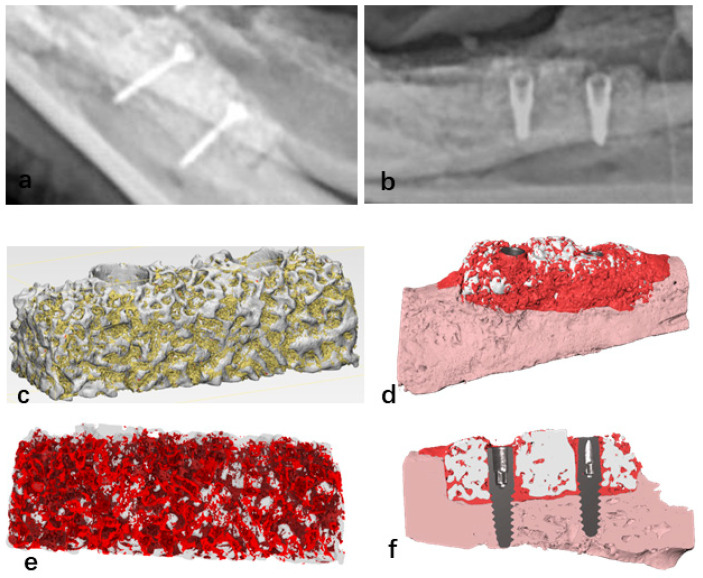
Bone healing image observed between the bioactive glass scaffold and the bottom of the bone defect area (**a**); bone “integration” image observed between the implant and the bioactive glass scaffold 12 weeks after implant placement (**b**); micro-CT reconstruction images of bioactive glass scaffolds, where yellow indicates newly formed bone tissue and white represents scaffold materials (**c**); the stent was modified to be transparent in order to enhance the visibility of the newly formed bone tissue marked in red (**e**); micro-CT scan reconstruction image of the bioactive glass scaffold with dental implants, where white indicates the scaffold material, dark red signifies new bone tissue, light pink represents autologous alveolar bone tissue, and gray denotes the metallic implant (**d**,**f**).

X-ray scans revealed that exposed scaffold materials presented a low-density image at the bottom of the bone defect area. In the non-exposed bone grafts, radiographic images showed bone healing between the scaffold material base and the alveolar bone. Additionally, 12 weeks after implant placement, X-ray images showed bone healing between the implant and the bioactive glass scaffold (Figure 7a,b). Micro-CT scanning of the surgical specimens showed the presence of scattered newly formed bone tissue in the non-exposed scaffold materials. Twelve weeks post-surgery, micro-CT scanning of the A-W BG scaffold implanted in the mandibular bone defect area revealed that newly formed bone tissue was evenly distributed in both the mesial and distal directions, with an average bone growth height of 4.05 ± 0.55 mm. Specific analysis regions, referred to as regions of interest (ROI), are clearly defined within the reconstructed three-dimensional digital image model. The total volume of the ROI (i.e., benchmark 100%) serves as the denominator for calculating the bone volume fraction (Figure 7c). Subsequently, differentiate bone tissue from residual material based on Hounsfield Units (HU), with the aid of fluorescence labeling to identify newly formed bone tissue ((Figure 7e). Finally, calculate the fraction of newly formed bone volume using the following formula: BV/TV%. Micro-CT analysis showed that the proportion of newly formed bone tissue was 43.93 ± 4.68%.

### 4.4. Histopathological Morphological Evaluation

**Figure 8:**
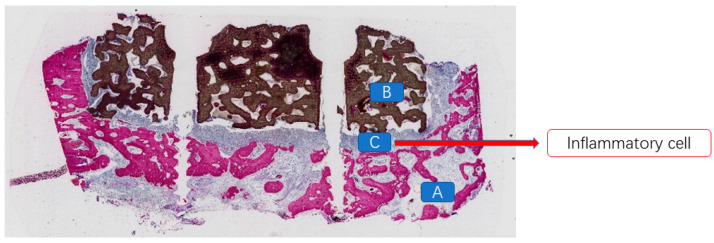
A large number of inflammatory cells present between the bioceramic scaffold and the alveolar bone in the exposed specimen. (A: alveolar bone; B: bioceramic scaffold; C: inflammatory cells).

**Figure 9:**
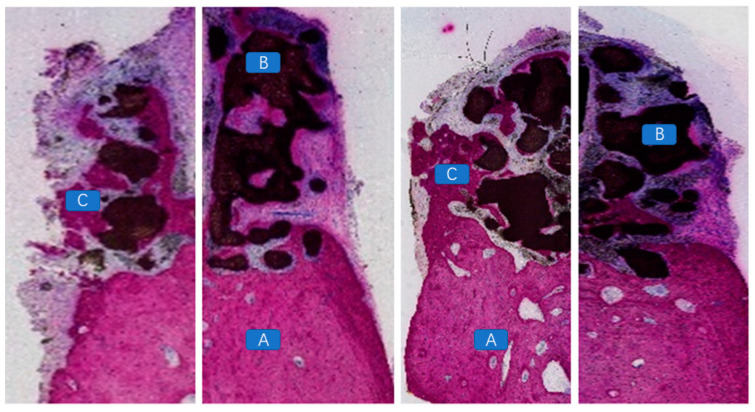
Vascular tissues, unmineralized bone, and newly formed mineralized bone observed between the scaffold material and the alveolar bone defect area, as well as within the scaffold material, in the non-exposed specimen. (A: alveolar bone; B: bioceramic scaffold; C: vascular tissues, unmineralized bone, and newly formed mineralized bone).

**Figure 10:**
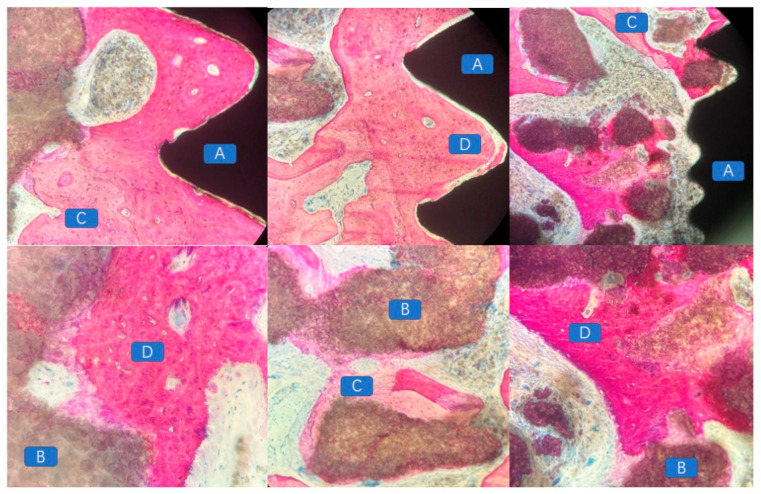
Histopathological analysis demonstrating the presence of newly formed bone tissue between the bioactive glass scaffold and the alveolar bone defect area, with a substantial amount of newly formed mineralized bone within the scaffold. Additionally, newly formed vascular and mineralized bone tissues were observed between the implant and the scaffold, indicating signs of “osseointegration”. (A: implant; B: residual tissue degraded by scaffold material; C: new uncalcified bone tissue; D: calcified mature bone tissue).

At 12 weeks postoperatively, in the mandibular specimens of Beagle dogs No. 01 and 03, a specific inflammatory response associated with a foreign body reaction was observed between the scaffold material and the bone defect area in the exposed specimens. A substantial presence of inflammatory cells and soft tissue was evident (Figure 8). In contrast, in the non-exposed specimens, vascular tissues, unmineralized bone, and newly formed mineralized bone tissues were distinctly observed between the scaffold material and the bone bed, as well as within the scaffold itself (Figure 9). At 12 weeks post-implantation in Beagle dogs, a histopathological analysis revealed the presence of newly formed bone tissue between the bioactive glass scaffold and the alveolar bone defect area. Additionally, a substantial amount of newly formed mineralized bone tissue was observed within the bioactive glass scaffold. Furthermore, newly formed vascular tissue and mineralized bone tissue were identified between the implanted fixture and the scaffold material, exhibiting signs of “osseointegration” (Figure 10).

## 5. Discussion

The reconstruction of severely atrophic alveolar ridges in implant regions presents a significant clinical challenge. Autologous bone blocks, bone substitute graft granules, and titanium mesh—commonly used scaffold materials—exhibit certain drawbacks. Therefore, the design of scaffold materials that integrate timely degradability, optimal spatial stability, osteoinductive properties, and the ability to restore individualized bone defect morphology in esthetic regions remains an ideal research objective. Additionally, whether the implanted scaffold material can subsequently support successful implant placement and achieve osseointegration comparable to autologous bone has yet to be investigated.

In this study, a 3D-printed bioactive glass scaffold was utilized to reconstruct large mandibular bone defects in Beagle dogs, followed by the first-time placement of implants. At three months postoperatively, osseointegration between the implant and the “ceramic bone block” was observed.

The A-W bioactive glass–ceramic used in this study has been previously reported in research concerning its mechanical strength and biological properties in guiding alveolar bone defect regeneration [32,33]. Since the regenerated bone region ultimately bears masticatory loads, sufficient mechanical strength is of critical importance. The mechanical properties of 3D-printed bioceramic scaffolds are influenced by their porosity, composite reinforcement materials, and material filling orientation [34,35]. In this study, a universal testing machine (SHIMADZU AGS-X, Kyoto, Japan) was employed to evaluate the mechanical compressive properties of the scaffold at a controlled room temperature of 25 °C. A total of six samples were tested, and the compression test of the homogeneously dense A-W BG scaffold yielded a compressive strength of 24.77 ± 2.36 MPa, with a coefficient of variation of 9.5246%, demonstrating favorable mechanical compressive performance.

**Figure 11:**
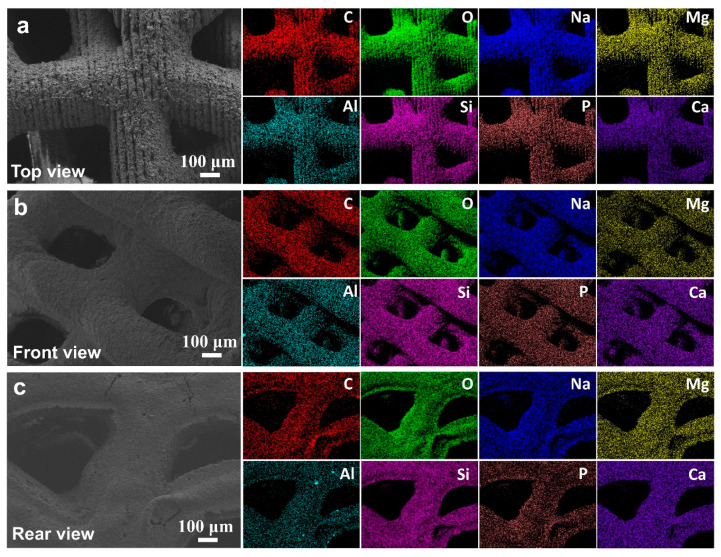
The morphology of the material under different viewing angles, along with the corresponding elemental energy-dispersive spectroscopy (EDS) results, where C, O, Na, Mg, Al, Si, P, and Ca are labeled in red, green, blue, yellow, cyan, pink, brown, and purple, respectively. (**a**) Top view, (**b**) Front view, (**c**) Rear view.

Figure 11 shows the multicolor EDS elemental mapping results, delineating the spatial distribution of C (red), O (green), Na (blue), Mg (yellow), Al (cyan), Si (pink), P (brown), and Ca (purple). The homogeneous dispersion of Si and Ca throughout the scaffold matrix supports the formation of a stable silicate network, while localized Mg and Al enrichment at pore boundaries suggests strategic doping to enhance mechanical strength without compromising bioactivity—a methodology paralleled in Harvard/Wyss Institute’s “compositionally graded scaffolds”. Notably, the absence of elemental segregation implies precise control over the 3D printing parameters (e.g., layer-by-layer deposition temperature, binder saturation). The synergistic integration of multiscale porosity and tailored elemental stoichiometry positions this scaffold as a competitive candidate for load-bearing bone repair, addressing the current limitations of brittleness and slow osseointegration rates observed in conventional bioactive glasses.

In guided bone regeneration for severely atrophic alveolar bone, autologous bone or bone substitute materials are commonly used in clinical practice to increase bone width and height, thereby preventing further resorption of the alveolar bone [36,37,38]. Studies comparing the effects of block autologous bone and particulate allogeneic bone in guided alveolar bone regeneration have demonstrated that block autologous bone grafts generally exhibit lower resorption rates and better maintenance of bone contour morphology [11,39,40]. Particularly in cases requiring extensive alveolar bone augmentation, research has further confirmed that block bone substitutes provide more effective support for surrounding structures and restore alveolar bone morphology to meet the requirements for implant placement compared to particulate substitutes [41]. Nevertheless, block bone grafts also have limitations, primarily due to their inability to conform precisely to the shape of bone defects, which may result in bone resorption and compromised osteogenesis [42,43,44]. The application of 3D printing technology allows for the preoperative customization of block bone grafts with predetermined dimensions and shapes based on the specific characteristics of the alveolar bone defect area [45,46], effectively addressing this issue. The key factor in 3D-printed scaffold materials lies in the selection of appropriate materials.

Medical bioceramic materials have been increasingly recognized as one of the most effective materials for guided bone regeneration due to their biocompatibility and excellent osteoconductive or osteoinductive properties [21,31,47]. Most bioceramic scaffolds are manufactured using conventional methods, such as gas foaming, fiber bonding, freeze-drying, phase separation/conversion, and particle leaching [48]. However, these methods do not allow for precise control over the pore shape, geometry, porosity, and interconnectivity of bioceramic scaffolds, nor do they enable the fabrication of scaffolds specifically designed to promote cell growth and tissue regeneration [49]. The use of 3D printing technology for the fabrication of personalized bioceramic scaffolds has further mitigated the limitations of traditional manufacturing methods [50,51].

Currently, various 3D printing systems are employed in the biomedical field. In previous studies, fused deposition modeling (FDM) 3D printing has been widely used to fabricate bone scaffold materials [52,53]. This technique involves mixing thermoplastic polymers with biomaterials and extruding them through a nozzle with a diameter of 0.4–0.5 mm to build the structure layer by layer. However, this method presents two major issues: first, it lacks the precision required for the detailed printing of osteogenic materials; second, the degradation of the incorporated polymers releases acidic byproducts, which often lead to tissue necrosis and failure of the bioceramic scaffold [54,55].

In this study, DLP 3D printing technology was employed, which represents one of the most advanced techniques in bone tissue engineering. This method enables the fabrication of biomaterials with a high resolution of 100 µm [26], utilizing light exposure to cure each deposited layer, thereby constructing complex tissue structures. This approach not only significantly reduces printing time, but also offers high precision in simulating bone defects [56,57]. Multiple studies have demonstrated that bioceramic scaffolds fabricated using DLP technology, such as calcium silicate bioceramics, hydroxyapatite (HA), and β-tricalcium phosphate (β-TCP) bioceramics, can be successfully applied in guided bone regeneration [31,58,59]. Additionally, researchers have modified the surfaces of bioceramic scaffolds produced via DLP technology by incorporating calcium phosphate powders or bioactive factors, which have been shown to enhance biological properties [60].

However, in this study, despite the absence of surface modifications or the addition of bioactive factors, successful new bone formation and osseointegration between the implant and scaffold material were achieved. A notable challenge in applying DLP technology is the photopolymerization of each material layer. Since traditionally used HA and TCP are not light-sensitive, they often need to be mixed with polymers for printing [61], followed by heat treatment to remove the polymer [62], making the process highly complex.

The consumable materials used in 3D printing constitute a fundamental prerequisite for the advancement of 3D printing technology. The materials available for 3D printing can be broadly categorized into four types: (1) polymer materials, such as plastics and rubber; (2) metallic materials, including aluminum alloys, titanium alloys, and stainless steel; (3) ceramic materials, such as gypsum, alumina, zirconia, and hydroxyapatite; and (4) derivative materials, comprising composite materials composed of tissue cells and hydrogels. Compared to polymers and metallic materials, ceramics exhibit inherent properties such as low toughness and high melting points, making them the most challenging materials to process using 3D printing technology. Scaffold materials for bone tissue engineering should possess favorable biocompatibility and biodegradability. The single-component bioceramic materials suitable for 3D printing include bioactive glass (BG), tricalcium phosphate (TCP), and hydroxyapatite (HA), all of which exhibit high biocompatibility comparable to that of conventionally manufactured products. However, due to their intrinsic brittleness, ceramics typically need to be compounded with alloys or polymers to achieve the mechanical properties required for optimal bone tissue engineering scaffolds.

In this study, the 3D-printed material utilized was an A-W bioactive glass–ceramic scaffold, classified as a bioactive ceramic. This material has been reported for its applications in bone defects, periodontal defects, and middle-ear reconstruction, as well as in vascular, tracheal, urethral, and skin repair [63,64]. The most distinctive characteristic of bioactive ceramics is their ability to form chemical bonds with the surrounding host tissue upon implantation, thereby eliminating the presence of a fibrous encapsulation layer between the material and bone, which facilitates firm fixation and effective bone repair. Furthermore, bioactive ceramics exhibit osteoconductivity, meaning that they promote new bone formation following implantation, thereby outperforming bioinert ceramics in osteogenic properties [65].

Bioactive ceramics have emerged as a significant focus in biomedical research and are regarded as second-generation biomedical materials. However, their primary drawback lies in their low flexural strength, high brittleness, and susceptibility to fracture, which limits their application as load-bearing implants. Currently, bioactive ceramics are primarily utilized in porous, block, and particulate forms for filling bone defects in non-load-bearing sites. Bioactive glass–ceramic materials have been developed as an improved alternative to conventional bioactive ceramics to address their limitations in mechanical strength and load-bearing capacity. This novel class of bioactive materials, also referred to as glass–ceramics, is represented by the A-W bioactive glass–ceramic utilized in this study.

In 1982, Kokubo T. et al. [66] developed a CaO-MgO-SiO_2_-P_2_O_5_-F-based glass–ceramic system, in which the primary crystalline phase was oxyfluoroapatite. The presence of oxyfluoroapatite microcrystals in the material enhances its bioactivity, while a large number of randomly oriented and uniformly distributed needle-like wollastonite crystals significantly improve its mechanical strength and machinability. In vitro experiments have demonstrated that, after immersion in simulated body fluid (SBF) for seven days, the surface of A-W bioactive glass–ceramic becomes covered with a hydroxyapatite layer. This phenomenon is attributed to the release of Ca^2+^ and HSiO_3_^−^ ions from the material, which play a crucial role in hydroxyapatite formation [67].

In vivo implantation experiments have shown that A-W bioactive glass–ceramic forms a strong chemical bond with bone after eight weeks of implantation in rabbit tibiae. Separation experiments conducted on rabbit tibial specimens implanted with the A-W bioactive glass–ceramic revealed that fractures occurred on the bone side rather than at the bone–material interface, indicating an exceptionally robust bond between the two [66].

In this study, bioactive glass scaffolds were implanted into the bone defect regions of Beagle dogs. Three months postoperatively, histopathological examination of the harvested surgical specimens revealed the presence of newly formed bone tissue between the bioactive glass scaffold and the alveolar bone defect region. Additionally, no mobility of the bioactive glass scaffold was observed under microscopic examination.

The experimental model in this study was established in the region extending from the premolars to the first molar of the mandible in Beagle dogs. Various animal models have been employed for guided bone regeneration in alveolar bone defects, including dogs [30,31,68], rabbits [58], and rats [69]. Among these, dogs are more widely used in dental and bone tissue regeneration research due to the anatomical similarities between their alveolar bone and that of humans [70]. Furthermore, the morphology and dimensions of the bone defect were designed with strict criteria. Although no definitive consensus has been reached regarding the critical size of bone defects in Beagle dogs, previous studies have demonstrated that guided bone regeneration can be successfully achieved in mandibular models featuring rectangular defects of 3 × 5 mm or box-shaped defects of 8 × 12 mm [71,72]. Excessively large defects may increase the risk of mandibular fractures, hemorrhage from the inferior alveolar vessels, and postoperative infection. In this study, the defect size was set at 15 × 5 × 5 mm. At 12 weeks postoperatively, flap exposure of the implanted bone defect scaffolds revealed that the scaffolds remained structurally intact, closely maintaining the original alveolar bone contour. A histopathological examination of scaffold specimens demonstrated the presence of vascular structures and newly formed bone within the scaffolds. Throughout this process, preserving the integrity of the periosteum was crucial for new bone formation [73], and, in the present surgical procedures, the periosteum of the canine mandible was preserved intact.

Furthermore, this preliminary study aimed to evaluate the effectiveness of 3D-printed bioactive glass–ceramic scaffolds in promoting bone augmentation in severe alveolar bone defects in Beagle dogs. Eight 3D-printed bioactive glass scaffolds were placed bilaterally in mandibular defects, and radiographic and histopathological evaluations were conducted three months after implantation. A histopathological analysis revealed newly formed bone tissue between the uninfected bioactive glass scaffold and the alveolar bone defect, as well as extensive newly formed calcified bone within the scaffold. Additionally, neovascularization and newly formed calcified bone tissue were observed at the interface between the implanted material and the scaffolds, with indications of osseointegration. Micro-CT analysis demonstrated a uniform distribution of newly formed bone tissue along the mesiodistal direction within the scaffolds, with an average bone height of 4.05 ± 0.55 mm. The proportion of newly formed bone tissue within the scaffold was 43.93 ± 4.68% based on the micro-CT analysis. Ju-Won Kim et al. conducted a guided bone formation experiment on the mandibular defect of dogs with a HA/TCP biological scaffold made using DLP 3D printing technology and found that the new bone formation volume fraction was 41.08 ± 3.96% at 8 weeks after surgery. It seems that, under the same experimental conditions, the bioactive glass scaffold material can produce a better osteogenic effect.

Finally, the problems of stent material exposure and infection in this experiment have to be faced and discussed. Firstly, the problems of doctors and aseptic operation were basically excluded because we carried out the relevant operations strictly. There are two main reasons for the exposure and infection of materials in the experiment. First, the tension of gingival tissue increased after the implantation of the scaffold material; second, the porosity of the material itself led to bacterial colonization, and the material had no anti-infection ability, resulting in wound infection. We propose two expected solutions based on the causes and plan to conduct follow-up experiments. We considered using a bio-collagen membrane to cover the surface of the scaffold material, which can act as a barrier for oral microorganisms. On the other hand, to enhance the inherent antibacterial properties of the material, strategic approaches may involve either engineering controlled release of antimicrobial agents during its degradation phase or incorporating antibacterial compounds within the material’s porous structure. This dual modification strategy significantly amplifies antimicrobial efficacy while maintaining material functionality.

By establishing a bone defect model in the mandible of Beagle dogs, this study preliminarily assessed the performance, safety, and osteogenic efficacy of 3D-printed bioceramic scaffolds in guiding alveolar bone regeneration using radiographic and histopathological methods. Additionally, for the first time, the osseointegration of implants inserted into newly formed bone within the bioceramic scaffolds was evaluated.

## 6. Conclusions

In this study, the application of A-W bioactive glass–ceramic scaffolds, fabricated using a DLP 3D printer, for the regeneration of severely deficient alveolar bone represents an innovative approach. This technology enables the fabrication of customized “autologous-like bone blocks” that precisely match the morphology of alveolar bone defects. However, scaffold material exposure was the primary challenge encountered, which may have influenced the mechanical strength and bone regeneration capacity of the 3D-printed bioactive glass scaffolds. Nonetheless, in successfully treated cases, complete bone healing was achieved between the bioactive glass scaffolds and alveolar bone defects, along with successful implant placement and osseointegration. These findings suggest that the application of this material in the regeneration of severely deficient bone is a feasible approach.

## Data Availability

The original contributions presented in this study are included in the article and; further inquiries can be directed to the corresponding authors.
